# Psychological Safety and Burnout in Nurses: A Scoping Review

**DOI:** 10.7759/cureus.92411

**Published:** 2025-09-15

**Authors:** Tomoo Sato, Kyosuke Kakuda, Eri Sekiguchi, Mitsuko Ishiseki, Mutsumi Iwanami, Yukie Akamatsu, Shunsuke Taito

**Affiliations:** 1 Division of Acute Care Nursing, Kobe City College of Nursing, Kobe, JPN; 2 Division of Psychiatric and Mental Health Nursing, Kobe City College of Nursing, Kobe, JPN; 3 Division of Child Health Care Nursing, Kobe City College of Nursing, Kobe, JPN; 4 Division of Nursing Theory and Practice, Kobe City College of Nursing, Kobe, JPN; 5 Division of Rehabilitation, Hiroshima University Hospital, Hiroshima, JPN

**Keywords:** psychological safety, scoping review, staff burnout, staff nurse, workplace environment

## Abstract

Burnout among nurses has become a global problem, with prevalence rates exceeding 40% in high-intensity clinical settings. Psychological safety represents a shared belief about team interpersonal risk-taking safety. These constructs have emerged as potential protective factors in recent research. However, studies that simultaneously examine both constructs remain limited, thereby hindering the development of evidence-based interventions aimed at promoting psychological safety and preventing burnout.Therefore, we conducted a scoping review to evaluate and synthesize existing literature that investigates the direct relationship between psychological safety and burnout among nurses, with emphasis on the conceptualization, measurement, and contextual association of these constructs within the nursing profession. This scoping review was conducted in accordance with the Joanna Briggs Institute methodology and complied with the Preferred Reporting Items for Systematic Reviews and Meta-Analyses extension for Scoping Reviews guidelines. A comprehensive literature search was conducted across Medical Literature Analysis and Retrieval System Online, Cumulative Index to Nursing and Allied Health Literature, Cochrane Central Register of Controlled Trials, Excerpta Medica Database, International Clinical Trials Registry Platform, and ClinicalTrials.gov. The search was conducted using terms related to psychological safety, burnout, and nurses. Studies were deemed eligible if they involved nurses employed in hospital-based settings and simultaneously examined the concepts of psychological safety and burnout. All published studies that examined psychological safety and burnout among nurses were included, with data extracted on study design, setting, country of origin, publication year, definitions used, measurement instruments, reported scores, and associated factors. Of 1,021 initially identified studies, six met the inclusion criteria, comprising 4,984 nurses across the United States, China, Pakistan, Japan, and South Korea, published between 2021 and 2025. Study designs included four cross-sectional studies, one longitudinal study, and one non-randomized controlled trial, conducted across diverse healthcare settings, including coronavirus disease 2019 wards, emergency departments, and psychiatric units. Three studies reported a consistent inverse relationship between psychological safety and burnout. Psychological safety was consistently defined in accordance with Edmondson's framework, while burnout was assessed based on Maslach's three-dimensional conceptualization. Leadership style, particularly servant leadership, emerged as a key factor potentially influencing both constructs. Workplace conditions, including patient acuity, exposure to workplace violence, and team dynamics, were identified as significant contextual factors.This review provides evidence of a consistent inverse relationship between psychological safety and burnout among nurses, with Edmondson's and Maslach's frameworks offering appropriate conceptual foundations for future research. These findings suggest that promoting psychological safety serves as a protective organizational strategy in high-intensity clinical settings. They also underscore the need for comprehensive, multi-level interventions focused on leadership development, structured communication protocols, and robust organizational support systems to enhance psychological safety and mitigate the risk of burnout.

## Introduction and background

Burnout and psychological safety have gained increasing attention in healthcare research owing to their profound implications for staff well-being and patient outcomes. Psychological safety, conceptualized by Edmondson in 1999 as "a shared belief that the team is safe for interpersonal risk-taking" [1], fosters learning, creativity, and engagement among healthcare professionals [2]. This concept is increasingly recognized as critical in diverse team-based environments, particularly in sectors such as healthcare, education, and organizational labor, where interpersonal risks may hinder performance and well-being [2]. Burnout, characterized by emotional exhaustion, depersonalization, and diminished personal accomplishment [3], is highly prevalent among nurses, with meta-analyses reporting overall rates exceeding 40%, particularly in working intensive care units (ICUs) [4]. It correlates with emotional dysfunction, fatigue, sleep disturbances, cognitive impairment, job dissatisfaction, absenteeism, decreased work productivity, and an increased intention to leave the profession [5,6]. Hospital-based nurses are at heightened risk of experiencing burnout due to the emotional burden of caring for critically ill patients, frequent exposure to death, and the demands of high-stakes, time-sensitive clinical decisions.

Psychological safety and burnout outcomes are profoundly influenced by the workplace environment. Workplace bullying and perceived leadership injustice have been identified as significant contributors to burnout, job dissatisfaction, absenteeism, decreased productivity, and higher turnover among nurses [6,7]. Conversely, environments that foster psychological safety are associated with increased team engagement, job satisfaction, learning, creativity, and a lower rate of burnout and turnover [3,5]. Psychological safety is posited as a protective factor against burnout, highlighting the need to clarify the relationship between these two constructs [1]. The global shortage of nurses further intensifies these challenges, which is an issue significantly exacerbated by the coronavirus disease 2019 (COVID-19) pandemic. Therefore, securing an adequate nursing workforce has become a critical challenge, thereby highlighting the importance of understanding and addressing the relationship between psychological safety and burnout [8].

While psychological safety and burnout are increasingly recognized in healthcare settings, research investigating the relationship between these constructs within nursing populations remains limited. Most existing studies explore these constructs independently, with association addressing their potential interconnection [9]. This gap is particularly evident across diverse healthcare settings, where the specific stressors and emotional demands encountered by nurses may differentially influence the relationship between psychological safety and burnout [6]. However, the limited number of studies that simultaneously examine both constructs has led to an insufficient understanding of their interconnection. Although some evidence suggests that supportive leadership and cohesive team dynamics contribute to increased psychological safety and reduced burnout [10], the underlying mechanisms of this relationship remain insufficiently explored, particularly within high-pressure clinical environments.

Therefore, this scoping review aims to comprehensively map the existing body of literature that simultaneously examines both psychological safety and burnout among nurses. This review aims to identify and synthesize studies that examine the direct relationship between these constructs, guided by the Preferred Reporting Items for Systematic Reviews and Meta-Analyses extension for Scoping Reviews (PRISMA-ScR) framework and the Joanna Briggs Institute methodology. Furthermore, this review assesses how psychological safety and burnout are conceptualized and measured in tandem, documents reported associations, and describes contextual factors that influence their interrelationship within nursing settings.

## Review

Materials and methods

Protocol and Registration

In accordance with a predefined protocol [11], this scoping review was conducted following a five-stage framework outlined by the Joanna Briggs Institute. These stages include the following: identifying the research question; identifying relevant studies; study selection; data charting; and collating, summarizing, and reporting the results [12]. This scoping review was conducted in accordance with the PRISMA-ScR [13] guidelines. Comprehensive methodological details can be found in the scoping review protocol, which was prospectively registered on the Open Science Framework (OSF) on May 5, 2025 [11].

Eligibility Criteria and Search Strategy

Inclusion criteria were established based on the population, concept, and context framework, in accordance with the Joanna Briggs Institute recommendations [12]. Studies were deemed eligible for inclusion if they involved hospital-based nurses and examined both psychological safety and burnout, constructs within the nursing context. Nurses were included regardless of country of origin, follow-up duration, publication status, or language. Studies that did not align with the conceptual framework of the present review were excluded from the study. All published randomized controlled trials, including crossover, cluster-randomized, quasi-randomized, and non-randomized trials, controlled observational studies, cross-sectional studies, and cohort studies, were included in the analyses. Studies that included healthcare professionals other than nurses, without providing a stratified analysis specific to nurses, were excluded. Conference abstracts, editorials, reviews, opinion papers, and study protocols were excluded from the analysis. Studies exclusively targeting neonatal or pediatric ICU nurses were excluded due to varying occupational dynamics.

A comprehensive search of the following databases was conducted on May 2, 2025: Medical Literature Analysis and Retrieval System Online (MEDLINE) via PubMed, the Cumulative Index to Nursing and Allied Health Literature (CINAHL), and Cochrane Central Register of Controlled Trials (CENTRAL) and Excerpta Medica Database (Embase). International Clinical Trials Registry Platform (ICTRP) and  ClinicalTrials.gov were searched to identify ongoing clinical trials.

Selection of Sources of Evidence 

All identified records were imported into Rayyan (Rayyan Systems Inc., Cambridge, Massachusetts, United States), an online tool designed to facilitate the management of systematic reviews. Following the removal of duplicates, six reviewers (TS, KK, ES, MI, YA, and MI) independently screened the titles and abstracts for potential eligibility. Full-text articles were subsequently retrieved and evaluated for final inclusion based on the predefined eligibility criteria. Any discrepancies between the reviewers were resolved through discussion or consultation with a third reviewer (ST). The entire selection process was documented in accordance with the PRISMA-ScR flow diagram, detailing the number of records identified, screened, evaluated for eligibility, and included in the final review.

Data Charting Process

A standardized data charting form was constructed using Microsoft Excel (Microsoft Corporation, Redmond, Washington, United States) to extract relevant information from each included study. Two independent reviewers conducted data charting, with discrepancies resolved through discussion. The following variables were extracted: author(s), year of publication, country of origin, study design, setting, sample size and demographic characteristics, definitions, and measurement tools employed, and associated factors. Where necessary, the authors of the reviewed publications were contacted.

Results

Selection of Sources of Evidence

**Figure 1:**
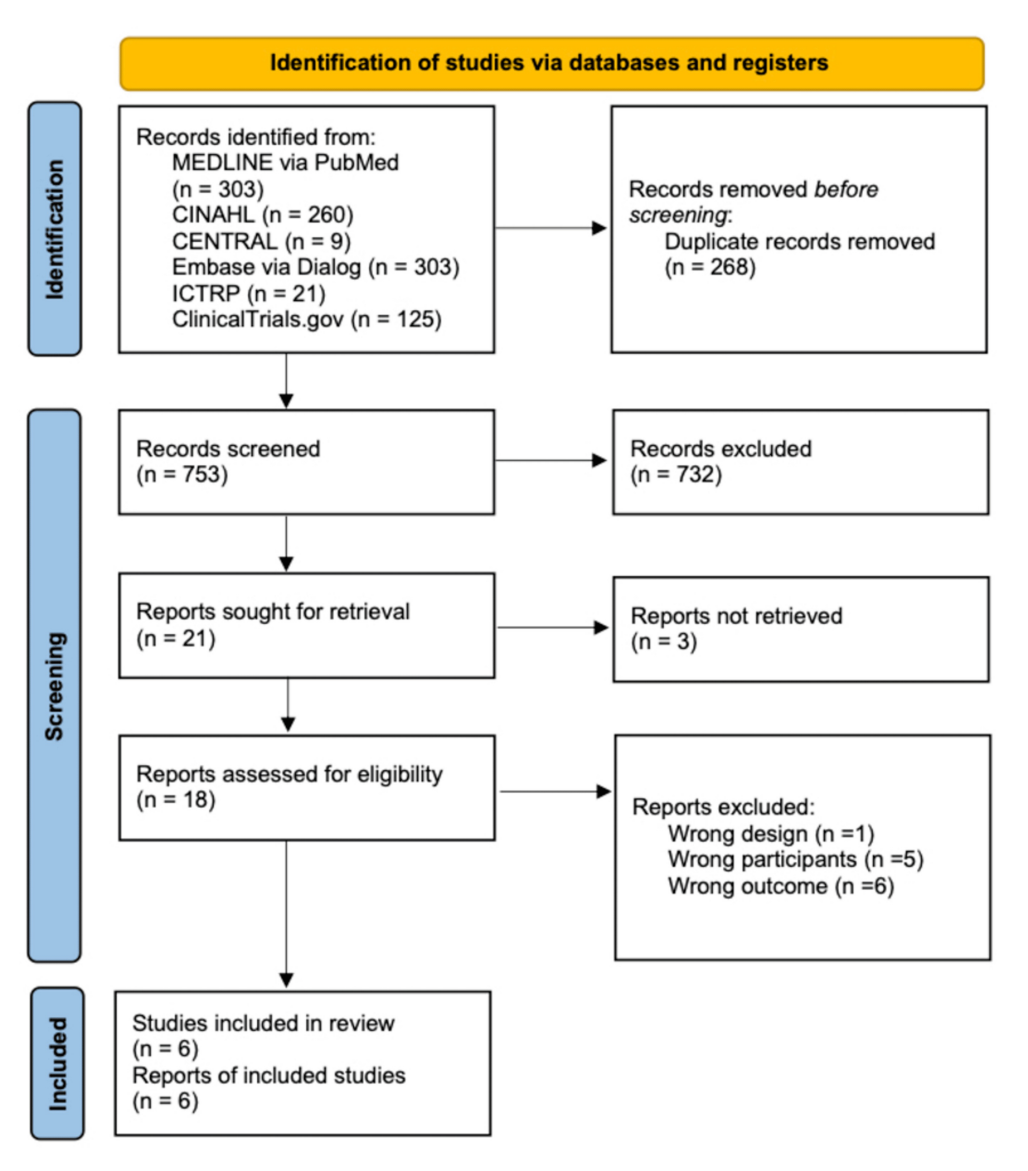
PRISMA flow diagram of the literature search results MEDLINE: Medical Literature Analysis and Retrieval System Online; CINAHL: Cumulative Index to Nursing and Allied Health Literature; CENTRAL: Cochrane Central Register of Controlled Trials; Embase: Excerpta Medica Database; ICTRP: International Clinical Trials Registry Platform; PRISMA: Preferred Reporting Items for Systematic Reviews and Meta-Analyses

Overall, 1,021 records were identified through six electronic databases and trial registries: MEDLINE via PubMed, CINAHL, CENTRAL, Embase, ICTRP, and ClinicalTrials.gov. Following the removal of 268 duplicates, 753 titles and abstracts were screened. Of these, 732 were excluded for not meeting the inclusion criteria. The full texts of 21 articles were retrieved for detailed evaluation; however, three could not be accessed. Among the 18 studies assessed for eligibility, 12 were excluded due to incorrect study design (n=1), inappropriate study population (n=5), or irrelevant outcomes (n=6). Ultimately, six studies met the eligibility criteria and were included in the final review, comprising 4,984 nurses [14-19]. The selection process is summarized in the PRISMA 2020 flow diagram (Figure 1).

Characteristics of the Included Studies

Table 1 presents a summary of the characteristics of the studies included in this review. The six studies were published between 2021 and 2025 and were conducted across various countries, including the United States [14,15], China [16], Pakistan [19], Japan [17], and South Korea [18]. All studies employed a quantitative research design: four were cross-sectional [14-16,18], one was longitudinal [16], and one was non-randomized controlled [17]. Sample sizes varied widely, ranging from 100 to 1,645 nurses. Two studies focused exclusively on COVID-19 wards [16,19], one on a private psychiatric ward [17], one on an emergency department [14], one on general and specialized units [18], and one on nurses in Patient-Aligned Care Team [14]. 

**Table 1:** Characteristics of the included studies COVID-19: coronavirus disease 2019; N/A: not available

Author	Year	Country	Study design	Setting	Participants	Definition of psychological safety	Definition of burnout
Ma et al. [19]	2021	Pakistan	Cross-sectional	COVID-19 wards	443	Edmondson	Maslach
Ahmed et al. [16]	2023	China	Longitudinal	Nurses providing direct care to patients with COVID-19	1,204	Edmondson	Maslach
Farag et al. [14]	2024	United States	Cross-sectional	Patient-Aligned Care Team members of nurses	1,645	N/A	Maslach
Lee et al. [18]	2024	South Korea	Cross-sectional	General (medical, surgical, or medical-surgical units) and specialized (intensive care units, perioperative units, and emergency departments) units	1,255	Edmondson	N/A
Kotake et al. [17]	2025	Japan	Non-randomized controlled trial	Private psychiatric hospital	100	Edmondson	N/A
Munn et al. [15]	2023	United States	Cross-sectional	Emergency department	337	Edmondson	N/A

Definitions of Psychological Safety and Burnout

The primary concepts addressed included psychological safety (n=5) [14-19] and burnout (n=3) [14,16,19] (Table 2). Definitions and measurement approaches for psychological safety and burnout varied across the six included studies but were generally aligned with established frameworks. Psychological safety was defined as "a shared belief that the team is safe for interpersonal risk-taking" [1], consistent with Edmondson's original conceptualization [14-19]. Burnout was defined according to Maslach [14,16,19] as a prolonged response to chronic interpersonal stressors in the workplace, characterized by emotional exhaustion, depersonalization, and a reduced sense of personal accomplishment. Burnout was commonly measured using the Maslach Burnout Inventory (MBI) [14-16,19], whereas psychological safety was assessed using instruments adapted from the original scale by Edmondson or from other validated instruments [16-19]. 

**Table 2:** Measurement tools and scores for psychological safety and burnout COVID-19: coronavirus disease 2019; EE: emotional exhaustion; DP: depersonalization; PA: personal accomplishment; N/A: not available; SD: standard deviation; PACT: Patient-Aligned Care Team; VHA: Veterans Health Administration; PTO: paid time off

Author, year	Psychological safety		Burnout		Psychological safety association factors		Burnout association factors	
	Measurement tool	Score (mean±SD)	Measurement tool	Score (mean±SD)	Positive correlation	Negative correlation	Positive correlation	Negative correlation
Ma et al. 2021 [19]	Detert and Burris's score (based on Edmondson's score)	4.58 (±0.92)	Maslach Burnout Inventory	4.73 (±0.87)	Servant leadership	Burnout	N/A	Servant leadership, psychological safety
Ahmed et al. 2023 [16]	Detert and Burris's score (based on Edmondson's score)	N/A	Maslach Burnout Inventory	N/A	Servant leadership	Burnout	N/A	Servant leadership, psychological safety
Farag et al. 2024 [14]	5-point Likert scale (7 items)	3.95 (±0.86)	Maslach Burnout Inventory	​​High burnout (≥3), followed by 40.12% of regular nurses ​	N/A	N/A	Tenure in the VHA and PACT; greater experience among the PACT members	Older PACT members, leadership support, quality team interaction, and psychological safety
Lee et al. 2024 [18]	Edmondson's score	N/A	Copenhagen Burnout Inventory	N/A	Perceived impact, voice	Silence, burnout	Silence	Perceived impact, psychological safety, and voice
Kotake et al. 2025 [17]	Edmondson's score	54.37 (±1.86)	Japanese Burnout Scale	EE: 2.91 (±0.14). DP: 2.15 (±0.13). PA: 3.65 (±0.11)	Trauma‐informed care video training	N/A	N/A	N/A
Munn et al. 2023 [15]	5-point Likert scale	N/A	Maslach Burnout Inventory	26.3 (±11.9)	N/A	N/A	ED volume, workplace violence (patient/visitor only), workplace violence (peer only), overtime hours worked, nurse staffing, total clinical staffing	Patient acuity, night shift, midday shift, perceptions of safety, PTO, inclusive culture, teamwork, well-being support, recognition, compensation, nurse manager support, overall perception of nurse manager, sufficient resources, and psychological safety

Measurement Tools

Measurement approaches for psychological safety and burnout varied across the six included studies but were generally aligned with established frameworks. Psychological safety was defined as "a shared belief that the team is safe for interpersonal risk-taking" [1], consistent with Edmondson's original conceptualization. Measurement instruments included Edmondson's original scale [16-19], with two studies utilizing versions that had been developed and translated into Korean [20]. Additionally, some studies employed adapted items rated on a 5-point Likert scale [18,19] to assess the freedom to express opinions, ask questions, or report errors without fear of negative consequences.

Burnout was frequently measured using the MBI [4], which conceptualizes burnout as comprising these three dimensions [14-16,19]. One study employed the Copenhagen Burnout Inventory (CBI) [21], which evaluates burnout across personal, work-related, and client-related domains. Two studies employed alternative burnout measurement instruments: Lee et al.'s study [18] used the CBI [21], which evaluates burnout across personal, work-related, and client-related domains, while Kotake et al.'s study [17] utilized the Japanese Burnout Scale [22].

Associations Between Psychological Safety and Burnout

Three studies [16,18,19] directly analyzed the relationship between psychological safety and burnout among nurses. All three reported statistically significant negative correlations, indicating that higher psychological safety levels were associated with lower levels of burnout. For instance, one cross-sectional study from Pakistan [19] found that greater perceived psychological safety levels were associated with lower burnout and increased work engagement (p<0.05). A South Korean study [18] employed structural equation modeling to demonstrate that psychological safety indirectly reduced burnout through mediating variables such as "voice" and "silence" behaviors, highlighting the importance of open communication climates. Additionally, a longitudinal study from China [16] demonstrated that servant leadership measured at time point 1 reduced burnout at time point 3, with psychological safety measured at an intermediate time point serving as a mediating variable.

Factors Associated With Psychological Safety and Burnout

Several key factors influencing psychological safety and burnout among nurses were identified. Leadership characteristics emerged as the most prominent, with servant leadership consistently associated with increased psychological safety and reduced burnout across multiple studies [16,17]. Environmental factors within the workspace also significantly influenced both constructs. High patient acuity, workplace violence from patients and visitors, and perceived physical insecurity were linked to greater levels of burnout and lower psychological safety [15]. Individual and demographic factors also played important roles. Variables such as age, tenure in healthcare organizations, and specific nursing roles (such as primary care providers) were associated with differing levels of psychological safety and burnout [14]. Communication behaviors functioned as important mediating mechanisms: "voice" behaviors (speaking up about concerns) were positively associated with psychological safety and negatively with burnout, while "silence" behaviors showed the opposite pattern [18]. Organizational support systems, including supervisors and peer support, nurse manager responsiveness, recognition programs, and adequate compensation, were consistently associated with higher psychological safety and lower burnout levels [14,15].

Discussion

This scoping review examined the current literature that concurrently addresses psychological safety and burnout among nurses, identifying six studies conducted between 2021 and 2025 across healthcare settings in five countries. These studies consistently reported a negative correlation between psychological safety and burnout, indicating that psychological safety functions as a protective factor against burnout in nursing practice. Key influencing factors were identified, including leadership styles, workplace environmental conditions, communication behaviors, and organizational support systems. These findings advance the understanding of the relationship between psychological safety and burnout and may inform the development of targeted interventions.

Definitions of Psychological Safety

The definition of psychological safety by Edmondson appears to provide the most suitable conceptual framework for nursing practice. Psychological safety was conceptualized by Kahn [23] and Edmondson [1], each from distinct perspectives. Kahn [23] defines psychological safety as the perception that one can express oneself without fear of negative consequences. In contrast, Edmondson [1] defines it as a shared belief among team members that the environment is safe for interpersonal risk-taking. Given the inherently collaborative and team-based nature of nursing practice, the group-level framework by Edmondson is particularly relevant. Five of the six studies explicitly defined psychological safety using Edmondson's framework [1], indicating conceptual consistency. This consistency suggests that, despite the relative novelty of the concept in nursing research, the field has converged on a standardized conceptual framework [24]. Measurement approaches were also largely consistent, with most studies employing the original scale by Edmondson [1] or validated adaptations, including Korean translations [25] and modified 5-point Likert scales. Ring and Hult [26] highlighted that perceptions of psychological safety may vary significantly across cultural contexts and organizational settings, suggesting that standardized instruments require cultural validation to ensure accurate assessment across diverse nursing populations. The consistent use of the definition by Edmondson offers a solid theoretical foundation for developing evidence-based interventions aimed at enhancing psychological safety in nursing environments. Therefore, healthcare organizations can implement psychological safety initiatives with confidence knowing they are anchored in a well-established conceptual framework [27]. Future studies should continue to utilize the definition by Edmondson to maintain consistency and facilitate meaningful comparisons across different healthcare settings and cultural contexts.

Definitions of Burnout

Maslach's three-dimensional model provides the most suitable conceptual framework for assessing burnout in nursing populations. All six included studies addressed burnout. Of these, four studies explicitly adopted Maslach's definition, which characterizes burnout as "a prolonged response to chronic interpersonal stressors in the workplace, characterized by emotional exhaustion, depersonalization, and reduced personal accomplishment" [4], or used the MBI, while the remaining two studies utilized other validated instruments for burnout. Although not all studies provided explicit definitions, the widespread use of the MBI suggests implicit adherence to the three-dimensional conceptualization by Maslach. One study utilized the CBI [21]. However, given that nursing is fundamentally a human services profession involving sustained interpersonal interactions with patients and families, the MBI remains the most appropriate instrument for this population. Recent empirical research provides additional insights into the dimensional structure of burnout. Taris et al. [28] indicated that elevated emotional exhaustion levels contribute to increased cynicism and depersonalization. Exhaustion and depersonalization represent the core dimensions of workplace burnout syndrome, whereas a lack of professional fulfillment may function either as an antecedent or as a consequence of the burnout process [29]. This hierarchical framework suggests that emotional exhaustion typically emerges first, leading to defensive psychological distancing. The widespread use of the MBI across studies facilitates meaningful comparisons and synthesis of findings, enabling healthcare organizations to monitor burnout levels and evaluate intervention effectiveness through validated, standardized metrics. Consequently, future research should continue to utilize the MBI as the standardized tool for measuring burnout among nurses, given its specific relevance to human services professions, while also investigating the longitudinal relationships between burnout dimensions to elucidate causal mechanisms and inform targeted intervention strategies.

Factors Associated With Psychological Safety and Burnout

Although studies on this topic remain limited, existing evidence indicates a negative correlation between psychological safety and burnout, with servant leadership emerging as a key factor. Three studies directly examined the relationship [16,18,19], all reporting statistically significant negative associations [16,18,19]. In a longitudinal study, Ahmed et al. [16] revealed that servant leadership mitigates burnout by enhancing psychological safety, suggesting a possible mediating mechanism. This finding aligns with the broader burnout literature, which emphasizes leadership styles as crucial organizational resources capable of mitigating job demands and preventing emotional exhaustion, the core dimension of burnout [10,17]. Additionally, this review found that the structural equation modeling conducted by Lee et al. [18] demonstrated an indirect effect of psychological safety on reducing burnout, mediated by increased voice behaviors and decreased silence, thereby highlighting communication as a key mechanism. This pathway aligns with existing evidence indicating that emotional exhaustion precedes cynicism and depersonalization [28], suggesting that psychological safety may interrupt this cascading process by promoting proactive problem-solving rather than defensive withdrawal. Other organizational factors included workplace environmental conditions (patient acuity, workplace violence, and physical security), communication behaviors, and support systems such as supervisor support. These factors align with well-established burnout antecedents, including excessive workload, insufficient supervision, and a lack of perceived social support [30]. Therefore, healthcare organizations should prioritize the development of servant leadership and creating psychologically safe environments as primary strategies for concurrently enhancing psychological safety and reducing burnout. Future research should employ intervention-based designs to examine whether psychological safety initiatives can prevent the sequential development of burnout dimensions and to determine the optimal timing for such interventions.

Limitations

Although the findings suggest consistent patterns across diverse contexts, some limitations should be acknowledged. First, most of the included studies employed cross-sectional designs, thereby limiting causal inferences. Second, the small number of included studies (n=6) and limited geographic diversity (primarily East Asia and the United States) restrict the generalizability of the findings.

## Conclusions

This scoping review demonstrates that psychological safety and burnout are consistently conceptualized using Edmondson's and Maslach's frameworks, respectively, with consistency in measurement approaches across studies. Three studies reported a significant negative correlation between psychological safety and burnout, with servant leadership identified as the primary protective factor influencing both constructs.

These findings support the role of psychological safety as a protective factor against burnout and establish a foundation for the development of targeted interventions within nursing environments. Future research should prioritize intervention-based designs to evaluate whether enhancing psychological safety can prevent burnout and to identify optimal strategies across diverse healthcare systems. 
